# Resveratrol production of a recombinant *Scheffersomyces stipitis* strain from molasses

**DOI:** 10.1016/j.biotno.2021.11.001

**Published:** 2021-12-02

**Authors:** Yuma Kobayashi, Kentaro Inokuma, Mami Matsuda, Akihiko Kondo, Tomohisa Hasunuma

**Affiliations:** a Graduate School of Science, Technology and Innovation, Kobe University, 1-1 Rokkodai-cho, Nada-ku, Kobe, 657-8501, Japan; b Engineering Biology Research Center, Kobe University, 1-1 Rokkodai-cho, Nada-ku, Kobe, 657-8501, Japan; c Biomass Engineering Program, RIKEN, 1-7-22 Suehiro-cho, Tsurumi-ku, Yokohama, Kanagawa, 230-0045, Japan

**Keywords:** *Scheffersomyces stipitis*, Non-conventional yeast, Resveratrol, Carbon catabolite repression, Molasses, Intracellular metabolite analysis

## Abstract

Resveratrol is a plant-derived aromatic compound with beneficial properties and it is required to develop a resveratrol production process from inexpensive biomass feedstocks. Here, we investigated the potential of *Scheffersomyces stipitis*, a non-conventional yeast with the capacity to utilize a wide range of sugars, to produce resveratrol from molasses, which is a by-product of sugar refineries. The *S. stipitis* strain metabolically engineered for resveratrol production produced resveratrol from 60 g/L mixed sugar (sucrose, glucose, and fructose), while its resveratrol titer decreased as the proportions of glucose and fructose increased. Sucrose consumption of the *S. stipitis* strain was clearly suppressed by the coexistence of glucose, fructose, and even ethanol. Quantitative analysis of intracellular metabolites involved in resveratrol biosynthesis using capillary electrophoresis time-of-flight mass spectrometry revealed that the composition of these sugars has a significant effect on the intracellular accumulation of glycolytic metabolites and AMP, which is an important factor involved in some cellular metabolic responses. Furthermore, the *S. stipitis* strain produced 1076 ± 167 mg/L of resveratrol in the fermentation with commercial sugarcane molasses (120 g/L of total sugars) as the substrate. To our knowledge, this is the first report on carbon catabolite repression in S*. stipitis* caused by the coexistence of sucrose, glucose, and fructose and resveratrol production from molasses. These results indicate great potential of the cost-effective resveratrol production process from molasses substrates using recombinant *S. stipitis*.

## 1 Introduction

**Fig. 1:**
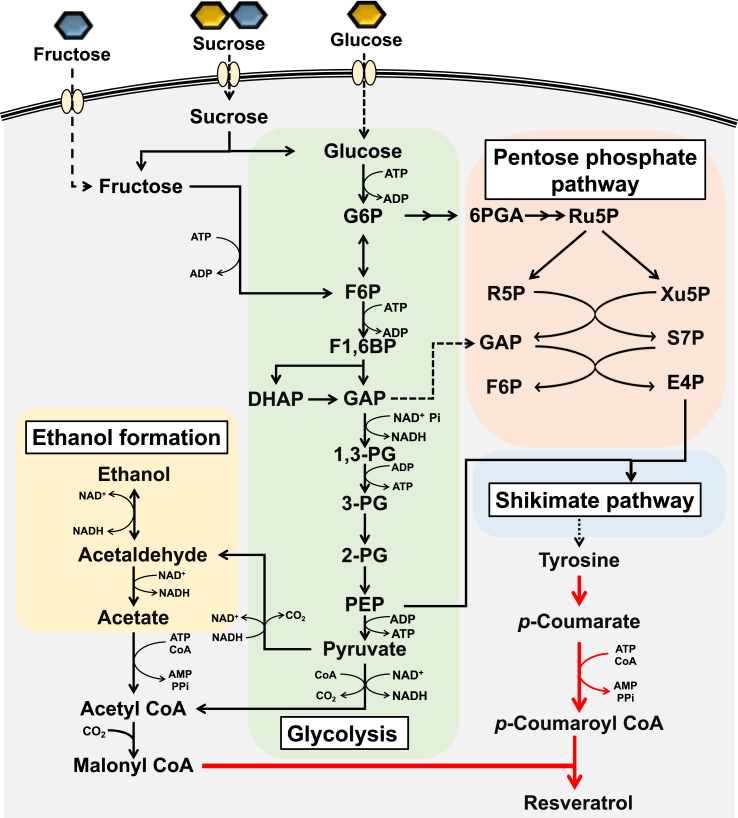
Schematic representation of resveratrol biosynthesis in the Ss-T4V-aro7m strain using sucrose, glucose, and fructose as carbon sources. Abbreviations: 1,3-PG, 1,3-bisphosphoglycerate; 2-PG, 2-phosphoglycerate; 3-PG, 3-phosphoglycerate; 6-PGA, 6-phosphogluconate; ADP, adenosine diphosphate; AMP, adenosine monophosphate; ATP, adenosine triphosphate; DHAP, dihydroxyacetone phosphate; E4P, erythrose-4-phosphate; F1,6BP, fructose-1,6-bisphosphate; F6P, fructose-6-phosphate; G6P, glucose-6-phosphate; NAD^+^, nicotinamide adenine dinucleotide; NADP^+^, nicotinamide adenine dinucleotide phosphate; PEP, phosphoenolpyruvate; PPi, pyrophosphate ion; Ru5P, ribulose-5-phosphate; R5P, ribose-5-phosphate; S7P, sedoheptulose-7-phosphate; Xu5P, xylulose-5-phosphate.

Resveratrol is a plant-derived aromatic compound known for its antioxidant and anti-aging properties^1^^,^^2^ and is used in dietary supplements, health foods, and cosmetics. Resveratrol and its derivatives are increasingly used as feed additives and functional polymers.^3^^,^^4^ Currently, several plants, such as the grape, peanut, and Japanese knotweed,^5^, ^6^, ^7^, ^8^ are major sources of commercial resveratrol. However, resveratrol extraction is expensive, and the purity of the yielded resveratrol is low.^5^^,^^7^ Therefore, cost-effective fermentation processes have been developed for the production of resveratrol from inexpensive sugars using recombinant microorganisms.^9^, ^10^, ^11^, ^12^ In the recombinant microorganisms, resveratrol is typically produced via three enzymatic reactions using the aromatic amino acid tyrosine as a precursor (Fig. 1). Tyrosine is deaminated by tyrosine ammonia lyase (TAL) to produce *p*-coumarate. *p*-Coumarate is then attached to coenzyme A (CoA) by 4-Coumarate: CoA ligase (4CL) to form *p*-coumaroyl-CoA. Finally, resveratrol synthase (VST) condensates one molecule of *p*-coumaroyl-CoA with three molecules of malonyl-CoA to synthesize resveratrol.

Yeasts such as *Saccharomyces cerevisiae* and *Yarrowia lipolytica* are promising host organisms for resveratrol production.^9^^,^^12^ Sáez-Sáez et al. (2020) reported the highest titer of resveratrol from glucose using metabolically engineered *Y. lipolytica* (titer, 12.4 g/L; yield, 54.4 mg/g glucose).^12^ Recently, we generated a novel resveratrol-producing strain of *Scheffersomyces stipitis*, a non-conventional yeast with the capacity to utilize a wide range of sugars. After introducing heterologous genes encoding TAL, 4CL, and VST and overexpressing an allele of chorismate mutase from *S. stipitis* (*SsARO7*^*G139S*^) that abolish allosteric feedback inhibition by tyrosine,^13^ the constructed *S. stipitis* strain successfully produced resveratrol from various biomass-derived sugars (glucose, fructose, xylose, *N*-acetyl glucosamine, galactose, cellobiose, maltose, and sucrose) as carbon sources. Additionally, this strain produced the highest resveratrol titer (668.6 mg/L) and yield (13.2 mg/g carbon source) with sucrose as the substrate, thereby suggesting that sucrose-containing feedstocks are attractive substrates for resveratrol production by the recombinant *S. stipitis*.^14^

A typical biomass-derived sucrose resource is waste molasses syrup, a viscous by-product of sugar refineries that is rich in sucrose (approximately 50% of dry weight).^15^ However, molasses also contains glucose and fructose, which are monosaccharides constituent of sucrose (13–25% of dry weight).^15^ The presence of different sugars often causes carbon catabolite repression and cellular metabolic changes, inhibiting the efficient production of target compounds by recombinant microorganisms.^16^, ^17^, ^18^ However, there are no reports of catabolite repression in *S. stipitis* caused by the coexistence of sucrose, glucose, and fructose, or of the impact of catabolite repression on the metabolic profile and resveratrol production.

Here, we evaluated the resveratrol-production capacity of a recombinant *S. stipitis* strain, Ss-T4V-aro7m, grown in mixed-sugar media comprising different proportions of sucrose, glucose, and fructose. We also quantitatively investigated the metabolic profile during fermentation using capillary electrophoresis time of flight mass spectrometry (CE-TOF-MS). Finally, we attempted the fermentation for resveratrol production in a medium containing sugarcane molasses as the carbon source.

## 2 Materials and methods

### 2.1 Yeast strains and media

The resveratrol-producing *S. stipitis* strain Ss-T4V-aro7m has been constructed by introducing genes involved in the resveratrol biosynthetic pathway [TAL from *Herpetosiphon aurantiacus* (*HaTAL1*), 4CL from *Arabidopsis thaliana* (*At4CL2*), and VST from *Vitis vinifera* (*VvVST1*)] and overexpressing a feedback inhibition-insensitive allele of chorismate mutase from *S. stipitis* (*SsARO7*^*G139S*^) in our previous study.^14^ Detailed genotype of the Ss-T4V-aro7m strain is shown in Table 1. This strain was cultivated in synthetic dextrose (SD) medium [6.7 g/L yeast nitrogen base without amino acids (Difco Laboratories, Detroit, MI, USA); 20 g/L glucose] at 30 °C for selection and pre-cultivation. Mixed sugar fermentation was performed in media containing 10 g/L yeast extract, 20 g/L peptone, and 60 g/L mixed sugar comprising sucrose and/or glucose and fructose at different proportions [60 g/L sucrose (S60); 40 g/L sucrose, 10 g/L glucose, and 10 g/L fructose (S40G10F10); 30 g/L sucrose, 15 g/L glucose, and 15 g/L fructose (S30G15F15); 20 g/L sucrose, 20 g/L glucose, and 20 g/L fructose (S20G20F20); and 30 g/L glucose, 30 g/L fructose (G30F30)].

**Table 1:** Yeast strains and plasmids.

Strains and plasmids	Relevant genotype	Ref
*Strains*		
*S. stipitis* NBRC10063	Wild type	NBRC
Ss102	*S. stipitis, ΔURA5, ΔADE2*	^14^
Ss-T4V-aro7m	Ss102{pInA2-T4V, pInU5-ARO7m}	^14^

*Plasmids*		
pInA2-T4V	*Amp*^*R*^*, ADE2, PIR1p-HaTAL1-TEF1t, ENO1p-At4CL2-UAGt, TEF1p-VvVST1-GLN1t*	^14^
pInU5-ARO7m	*Amp*^*R*^*, URA5, ENO1p-SsARO7*^*G139S*^*-UAGt*	^14^

For molasses-batch fermentation, sugarcane molasses (BMBio, Tokyo, Japan) containing 317 g/L sucrose, 148 g/L glucose, and 144 g/L fructose was used as the substrate. Fermentation tests were performed in a medium containing the molasses diluted to a total sugar concentration of 60 or 120 g/L, supplemented with 10 g/L yeast extract and 20 g/L peptone. The total sugar concentration was defined as the sum of the sucrose, glucose, and fructose concentrations.

### 2.2 Fermentation methods

Pre-cultivated yeast cells were inoculated into three independent 200 mL baffled flasks containing 30 mL of medium for each medium condition at an initial OD_600_ of 0.1. Fermentation was conducted at 30 °C in an orbital shaker incubator (100 rpm; BR-43FL; Taitec, Saitama, Japan), and the culture broths during fermentation were sampled for measurement of sugars, ethanol, and resveratrol concentrations by high-performance liquid chromatography (HPLC). Yeast cell growth in mixed sugar fermentation was measured as the optical densities of the culture medium at a wavelength of 600 nm (OD_600_). On the other hand, yeast cell growth in molasses-batch fermentation could not be measured because the fermentation media with molasses were dark in color and contained insoluble matter.

### 2.3 Extraction of intracellular metabolites

Samples for metabolome analysis were prepared as described previously.^19^ Briefly, 3 mL of the culture medium was sampled after fermentation for 24, 48, 72, and 96 h, and injected into a tube containing 7 mL of cold methanol immediately. Then leakage-free quenching^20^ was performed. Culture supernatant was removed by centrifugation, 10 μL of 40 mM 1,4-pip erazinediethanesulfonic acid (PIPES) and l-methionine sulfone were added to the samples as internal standards for the mass analysis of anionic and cationic species, respectively. Intracellular metabolites were extracted by boiling ethanol method,^21^ and the extracted metabolites were filtered through a 3-kDa molecular weight cut-off filter, dried in a vacuum evaporator overnight, and stored at −80 °C until use.

### 2.4 CE-TOF-MS analysis

The extracts were dried overnight using a vacuum evaporator (CVE-3100; Tokyo Rikakikai, Osaka, Japan) and stored at −80 °C until use. The dried metabolites were dissolved in ultrapure water, and the concentrations of the anionic and cationic intermediates were measured using CE-TOF-MS as previously described.^22^

## 3 Results

### 3.1 Mixed sugar fermentation

**Fig. 2:**
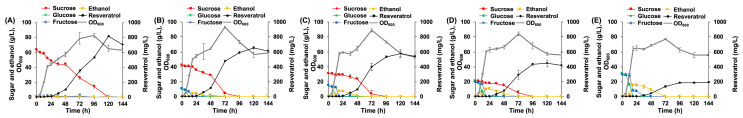
Fermentation with resveratrol-producing *S. stipitis* in mixed sugar media comprising sucrose, glucose, and fructose in different proportions. Fermentation tests were performed in media containing **(A)** 60 g/L sucrose (S60), **(B)** 40 g/L sucrose, 10 g/L glucose, and 10 g/L fructose (S40G10F10), **(C)** 30 g/L sucrose, 15 g/L glucose, and 15 g/L fructose (S30G15F15), **(D)** 20 g/L sucrose, 20 g/L glucose, and 20 g/L fructose (S20G20F20), and **(E)** 30 g/L glucose and 30 g/L fructose (G30F30), respectively. The data represent the mean ± standard deviation of three independent experiments.

To evaluate the fermentative behavior of the resveratrol-producing *S. stipitis* strain Ss-T4V-aro7m in mixed-sugar media, fermentation with different proportions of sucrose, glucose, and fructose was conducted using this strain. In the S60 medium, Ss-T4V-aro7m constitutively consumed sucrose and produced resveratrol throughout 120 h of fermentation, and no significant ethanol accumulation was detected (Fig. 2A). Overall, the resveratrol titer in the S60 medium reached 816 ± 4 mg/L, with a yield of 12.7 ± 0.2 mg/g sugar (Table 2). In contrast, in the three media containing glucose, fructose, and sucrose, glucose consumption occurred first, followed by fructose consumption and ethanol production (Fig. 2B–E). The ethanol produced was subsequently consumed after the depletion of glucose and fructose. Sucrose consumption was strictly suppressed until ethanol accumulation was eliminated (Fig. 2B–D). Furthermore, the resveratrol titer decreased as the proportions of glucose and fructose increased, dropping to 184 ± 12 mg/L in the medium containing only glucose and fructose (G30F30) (Figs. 2), 22.3% of that in the S60 medium, suggesting that the efficiency of resveratrol production from glucose and fructose was lower than that from sucrose.

**Table 2:** Sugar composition and resveratrol production in the mixed sugar fermentation.

Medium	Initial sugar composition			Maximum ethanol concentration (g/L)	Resveratrol production^a^	
	Sucrose (g/L)	Glucose (g/L)	Fructose (g/L)		Resveratrol titer (mg/L)	Yield (mg/g total sugar consumed)
S60	63.0 ± 1.2	–	–	0.8 ± 0.1	816 ± 4	12.7 ± 0.2
S40G10F10	41.8 ± 0.2	10.4 ± 0.2	10.7 ± 0.2	4.3 ± 0.1	658 ± 20	10.5 ± 0.3
S30G15F15	30.9 ± 0.5	15.8 ± 0.8	14.8 ± 0.9	7.5 ± 0.7	574 ± 39	9.4 ± 0.6
S20G20F20	20.8 ± 0.4	20.1 ± 0.6	19.1 ± 0.9	11.1 ± 0.8	446 ± 48	7.4 ± 0.8
G30F30	–	30.4 ± 0.5	28.9 ± 0.2	15.8 ± 0.3	184 ± 12	3.1 ± 0.2

a The values of resveratrol obtained from culture broths after 120 h fermentation.

### 3.2 Analysis of intracellular metabolites

To investigate the influence of mixed sugar conditions on the metabolic profile in each phase of fermentation, intracellular metabolites of Ss-T4V-aro7m grown in S60 (Fig. 2A), S40G10F10 (Fig. 2B), and G30F30 (Fig. 2E) media were extracted after 24, 48, 72, and 96 h of fermentation.

Sugar ratio of the S40G10F10 medium was relatively similar to that of sugarcane molasses.^15^

The extracted metabolites involved in resveratrol biosynthesis were measured by CE-TOF-MS, and 24 metabolites were detected in at least one of the samples. Concentrations [nmol/mg dry cell weight (DCW)] of the detected metabolites in each sample during fermentation are listed in Supplementary Table S1. Xylulose-5-phosphate (Xu5P), glyceraldehyde-3-phosphate (GAP), erythrose-4-phosphate (E4P), and malonyl-CoA were not detected in any of the samples.

In the early phase (within 48 h) of fermentation, most of the glycolytic metabolites, except pyruvate, accumulated more in cells cultivated in S60 medium than in those cultivated in S40G10F10 and G30F30 media. Furthermore, in the S60 medium, the accumulation of glycolytic metabolites decreased gradually during fermentation. In contrast, in the S40G10F10 and G30F30 media, the accumulation of glycolytic metabolites remained consistent or increased gradually.

**Fig. 3:**
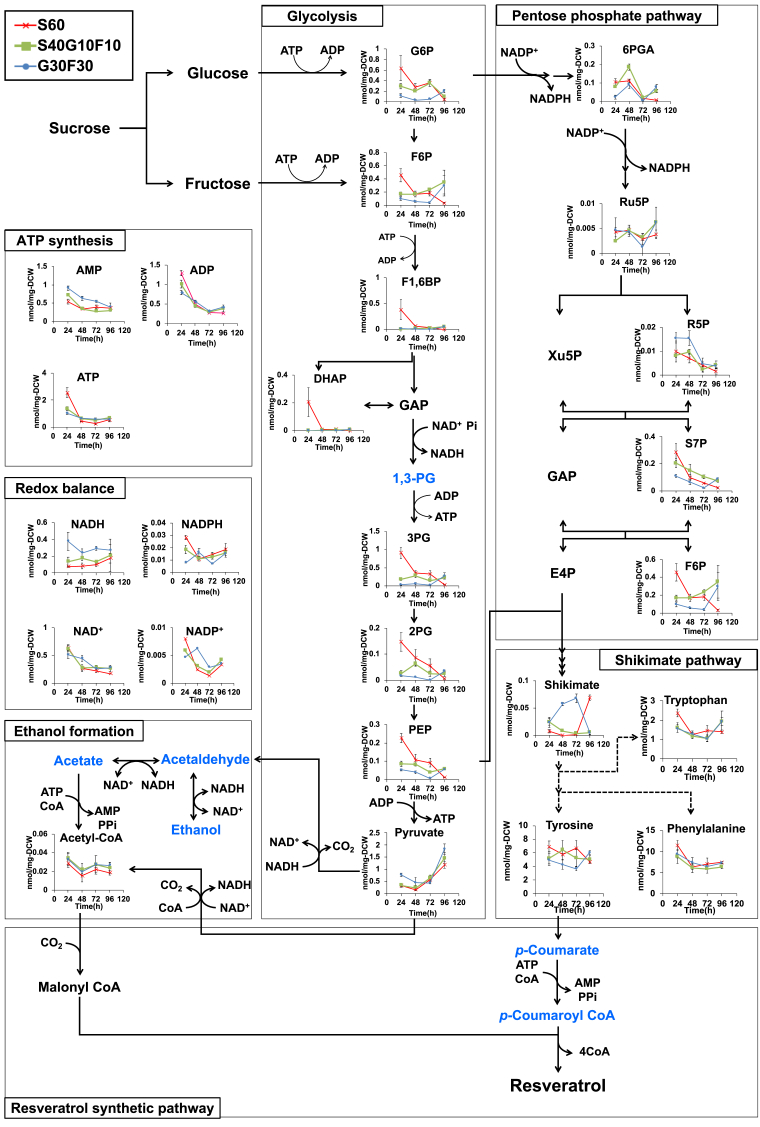
Time-course changes in the concentration of intracellular metabolites during fermentation in different mixed sugar media. Intracellular metabolite concentrations (nmol/mg DCW) of the Ss-T4V-aro7m strain grown in three different media, containing 60 g/L sucrose (S60 medium); 40 g/L sucrose, 10 g/L glucose, and 10 g/L fructose (S40G10F10 medium); and 30 g/L glucose and 30 g/L fructose (G30F30 medium); respectively. Metabolites written in blue were not analyzed in this study. Data are presented as the mean ± standard deviation (n = 3).

We also analyzed the intracellular accumulation of ATP and AMP during fermentation, because several reactions involved in resveratrol biosynthesis (CoA ligation of *p*-coumaric acid and acetate by 4CL and acetyl CoA synthase, respectively) are coupled with the conversion of ATP to AMP (Fig. 1). In the early phase (within 24 h) of fermentation, *S. stipitis* cells in S60 medium showed significantly greater ATP accumulation than those in S40G10F10 and G30F30 media (Fig. 3). Conversely, AMP accumulation was higher in G30F30 medium than in S60 and S40G10F10 media throughout fermentation.

### 3.3 Molasses fermentation for resveratrol production

**Fig. 4:**
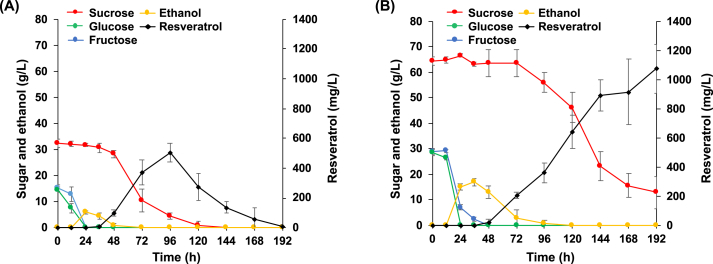
Molasses fermentation by the Ss-T4V-aro7m strain. Fermentation tests were performed in YP media with molasses composed of **(A)** 60 g/L and **(B)** 120 g/L of total sugars. The data represent the mean ± standard deviation of three independent experiments.

To verify the potential of the Ss-T4V-aro7m strain to directly produce resveratrol from biomass-derived feedstock containing sucrose, glucose, and fructose, fermentation tests were conducted using diluted commercial sugarcane molasses (60 or 120 g/L of total sugars) as a carbon source. Robust carbon catabolite repression was observed in the molasses fermentation (Fig. 4), similar to that in the mixed sugar fermentation (Fig. 2). In the fermentation with 60 g/L of total sugars (Fig. 4A), resveratrol production was started after 36 h. The resveratrol titer reached 504 ± 61 mg/L after 96 h when sucrose was depleted, and then gradually decreased. In the fermentation with 120 g/L of total sugars (Fig. 4B), although the start of resveratrol production was slightly later than that in molasses fermentation with 60 g/L of total sugars, the Ss-T4V-aro7m strain continued to produce resveratrol thereafter. The final resveratrol titer reached 1076 ± 167 mg/L in 192 h of fermentation, at which point approximately 12.8 g/L of sucrose remained in the medium.

## 4 Discussion

In general, most biomass-derived feedstocks contain a few mixed sugars. For example, the hydrolysate of lignocellulosic biomass contains a mixture of sugars, mainly glucose and xylose.^23^ Molasses contains mainly sucrose, glucose, and fructose.^15^ Under the coexistence of different carbon sources, most microorganisms including yeasts, preferentially utilize certain carbon sources, such as glucose, and the uptake of other carbon sources into the cells are inhibited,^24^, ^25^, ^26^, ^27^, ^28^ which is called carbon catabolite repression. Since *S. stipitis* is well known for its high xylose fermentation capacity, several studies on its glucose-induced catabolite repression on xylose fermentation have been reported.^24^^,^^29^ To our knowledge, however, catabolite repression on sugars in molasses (glucose, fructose, and sucrose) has not been investigated in *S. stipitis*.

In the present study, we evaluated the resveratrol-production capacity of a recombinant *S. stipitis* strain (Ss-T4V-aro7m) under mixed sugar condition (glucose, fructose, and sucrose) and demonstrated for the first time the catabolite repression on these sugars in *S. stipitis*. Although the Ss-T4V-aro7m strain was able to produce resveratrol under mixed sugar conditions, its resveratrol titer decreased as the proportions of glucose and fructose increased (Fig. 2B–E), thereby indicating that the efficiency of resveratrol production from glucose and fructose was lower than that from sucrose, which was consistent with our previous study.^14^

During the fermentation, sucrose consumption was clearly suppressed by the coexistence of glucose, fructose, and even ethanol. In *S. cerevisiae*, glucose-induced catabolite repression uses three signaling pathways: inhibition of AMP-activated protein kinase (AMPK^Snf1^), activation of protein kinase A (PKA), and the regulation of expression and stability of transporter by casein kinases.^30^ Similar signaling pathways may be involved in glucose-induced catabolite repression on sucrose fermentation in *S. stipitis*. One the other hand, little is known about how carbon sources other than glucose induce catabolite repression in yeast.^31^ Further studies will be needed to elucidate the molecular mechanism of the catabolite repression induced by carbon sources including fructose and ethanol in *S. stipitis*.

In the media containing glucose and fructose, Ss-T4V-aro7m produced ethanol from these monosaccharides (Fig. 2B–E), while no significant ethanol and monosaccharide accumulation was observed in the medium with only sucrose as a fermentable sugar (S60 medium, Fig. 2A). Similar suppression of ethanol fermentation was also observed in the production of resveratrol from cellobiose using this strain in our previous study.^14^ It has been generally recognized that the alcoholic fermentation in Crabtree-negative yeasts, including *S. stipitis*, are induced by not external glucose but oxygen limitation.^32^^,^^33^ In contrast, the present results suggest the existence of an induction mechanism of the alcoholic fermentation by external glucose and/or fructose in *S. stipitis*. Further research is needed on the molecular mechanism of alcoholic fermentation induced by these monosaccharides.

Quantitative analysis of intracellular metabolites involved in resveratrol biosynthesis using CE-TOF-MS revealed the impact of catabolite repression on the metabolic profile in *S. stipitis*. When cultured in the S60 medium, yeast cells accumulated most of the glycolytic metabolites, except pyruvate, in the early phase (within 48 h) of fermentation, and gradually these accumulations were reduced throughout fermentation. Conversely, in the S40G10F10 and G30F30 media, most of the glycolytic metabolites were lower than those of yeast cell cultivated in the S60 medium in the early phase (24 h), while the accumulation of the glycolytic metabolites reached or exceed to those of yeast cell cultivated in the S60 medium in the later phase (72 h and/or 96 h), where glucose and fructose were exhausted. This phenomenon was similar with the metabolic shift from glycolysis to gluconeogenesis in *S. cerevisiae* induced after glucose starvation,^34^ implying the induction of gluconeogenesis in *S. stipitis* after glucose and fructose exhausts. Additionally, there was a remarkable difference in the intracellular AMP concentration in the resveratrol production phase depending on the sugar composition of the medium. The AMP concentration was lower in the media yielding high resveratrol production. This suggests two explanations for the increased resveratrol production. First, AMP may have inhibited the activity of CoA ligase enzymes, such as ACS and 4CL, by feedback inhibition.^35^^,^^36^ Second, the changes in the cellular responses mediated by the AMPK cascade, which is activated by elevated AMP levels, may have been involved in the production of resveratrol. AMPK^Snf1^ is conserved among eukaryotes, including yeast^37^^,^^38^ and has been reported to inhibit the activity of the acetyl-CoA carboxylase 1 (ACC1) enzyme, catalyzing carboxylation of cytosolic acetyl-CoA to form malonyl-CoA in *S. cerevisiae*.^39^ Resveratrol synthesis requires condensation of three molecules of malonyl-CoA with one molecule of *p*-coumaroyl-CoA. Therefore, intracellular malonyl-CoA availability has a great impact on the resveratrol production. AMP accumulation in the G30F30 medium might have suppressed the supply of malonyl-CoA, thereby causing a decrease in resveratrol productivity.

We also investigated the potential of the Ss-T4V-aro7m strain to directly produce resveratrol from a molasses substrate. In the molasses fermentation with 60 g/L of total sugars, this yeast consumed almost all the sugars in the molasses (32.4 g/L sucrose, 14.6 g/L glucose, and 15.1 g/L fructose, Table 3) and produced approximately 504 ± 61 mg/L of resveratrol after 96 h of fermentation (Fig. 4A), which is similar to that in the S30G15F15 medium (Fig. 2C). Moreover, the amount of resveratrol produced in the molasses fermentation was observed to decrease after the carbon sources depletion. Decrease in resveratrol after substrate depletion has been reported in the resveratrol production by the metabolically engineered *Corynebacterium glutamicum* by Braga et al. (2018).^40^ They suggested that this phenomenon is due to the strong tendency of resveratrol to oxidize or oligomerize by elevated dissolved oxygen concentration after substrate depletion. Some compounds in molasses (e.g., organic acids^15^) may increase the oxidative stress of the fermentation medium after the substrate depletion and promoted the oxidation and oligomerization of resveratrol. In contrast, this strain produced 1076 ± 167 mg/L of resveratrol in the molasses fermentation with 120 g/L of total sugars (Fig. 4B). This resveratrol titer is higher than that from glucose in fed-batch fermentation using recombinant *S. cerevisiae* (812 mg/L).^9^ Although it does not reach the highest titer in fed-batch fermentation using metabolically engineered *Y. lipolytica* (12.4 g/L),^12^ these results indicate that Ss-T4V-aro7m has great potential for the direct production of resveratrol from molasses substrate. In the present study, the molasses fermentation was stopped at 192 h due to the limited volume of fermented media (30 mL), while the resveratrol concentration still showed a continuously upward trend at 192 h in the molasses fermentation with 120 g/L of total sugars (Fig. 4B). Scaling up the fermented media and extending the fermentation period may further improve the resveratrol production from molasses.

**Table 3:** Sugar composition and resveratrol productivity in the molasses fermentation.

	Initial sugar composition			Resveratrol production	
	Sucrose (g/L)	Glucose (g/L)	Fructose (g/L)	Titer (mg/L)	Yield (mg/g total sugar consumed)
10-fold diluted (60 g/L of total sugar)	32.4 ± 1.4	14.6 ± 1.2	15.1 ± 1.0	504 ± 6^a^	8.1 ± 1.2^a^
5-fold diluted (120 g/L of total sugar)	64.4 ± 1.7	28.8 ± 1.1	28.7 ± 1.5	1076 ± 167^b^	9.9 ± 1.2^b^

a The values obtained from culture sample after 96 h fermentation.

b The values obtained from culture sample after 192 h fermentation.

On the other hand, carbon catabolite repression on sucrose was observed in the molasses fermentation, similar to that in the mixed sugar fermentation. This lead to a delayed sucrose consumption and resveratrol production. It has been reported that cane molasses has a large variation in sucrose, glucose, and fructose composition [39.2–67.3, 1.3–12.1, and 2.3–14.3% of dry matter (DM), respectively] depending on the region where it is procured.^15^ In addition, beet molasses has higher sucrose concentration (60.9% of DM on average) and lower glucose and fructose concentration (0.3% of DM on average for both) compared to cane molasses.^15^ Although we used a commercial sugarcane molasses with relatively high glucose and fructose content as the substrate in the present study, the use of molasses with low glucose and fructose content as the substrate may enable reduced carbon catabolite repression on sucrose and efficient resveratrol production by the recombinant *S. stipitis*.

In conclusion, we demonstrated a potential of the resveratrol production process from molasses substrates using recombinant *S. stipitis*. One the other hand, carbon catabolite repression on sucrose, a preferred carbon source for resveratrol production, induced by glucose, fructose, and ethanol in *S. stipitis* was also revealed. Genetic engineering of this strain to overcome the catabolite repression is required to improve the resveratrol productivity from molasses substrates using this yeast. Our intracellular metabolite data may inform the rational design of engineering strategies in the recombinant *S. stipitis*. In addition, since the sugar content of molasses has a large variation as mentioned above, it would be also important to select the molasses with low glucose and fructose content for further improvement of the resveratrol productivity.

## Declaration of competing interest

The authors declare that they have no competing interests with the contents of this article.
